# Baseline SII and SIRI identify immune-inflammatory susceptibility and improve risk stratification of early subclinical cardiac dysfunction during non-steroidal aromatase inhibitor therapy

**DOI:** 10.3389/fcvm.2026.1888328

**Published:** 2026-10-09

**Authors:** Cenyu Li, Zhengyu Tao, Yihan Wu, Chen Wu, Zi Wang, Xiaoning Wang, Huijuan Dai, Meng Jiang, Jun Pu

**Affiliations:** 1 Division of Cardiology, State Key Laboratory of Systems Medicine for Cancer, Renji Hospital, School of Medicine, Shanghai Jiao Tong University, Shanghai, China; 2 Shanghai Jiao Tong University School of Medicine, Shanghai, China; 3 School of Mathematical Sciences, Shanghai Jiao Tong University, Shanghai, China; 4 Department of Breast Surgery, Renji Hospital, School of Medicine, Shanghai Jiao Tong University, Shanghai, China

**Keywords:** cardiovascular surveillance, HR+ breast cancer, non-steroidal aromatase inhibitor, study-defined composite cardiac dysfunction, systemic immune-inflammation index (SII), systemic inflammation response index (SIRI)

## Abstract

**Background:**

Non-steroidal aromatase inhibitors (NSAIs) are a cornerstone of adjuvant endocrine therapy in postmenopausal women with hormone receptor-positive (HR+) breast cancer. Given their prolonged use in an aging population with increasing cardiovascular vulnerability, we investigated whether baseline systemic immune-inflammation index (SII) and systemic inflammation response index (SIRI) could identify pretreatment immune-inflammatory susceptibility to NSAI-related early subclinical cardiac dysfunction.

**Methods:**

This single-center retrospective cohort study included 269 postmenopausal women with HR + breast cancer who received letrozole or anastrozole between January 2020 and April 2024. The primary endpoint was study-defined composite cardiac dysfunction, defined as either an absolute decline in left ventricular ejection fraction (LVEF) of ≥10 percentage points from baseline or a relative increase in B-type natriuretic peptide (BNP) of ≥40% from baseline together with a follow-up BNP concentration ≥100 pg/mL. Baseline SII and SIRI were calculated from routine blood counts and analyzed as z-standardized natural log-transformed variables. Logistic regression, restricted cubic spline, receiver operating characteristic curve, decision curve, sensitivity, subgroup, and exploratory machine-learning-assisted risk-stratification analyses were performed.

**Results:**

Among 269 patients, 63 (23.4%) met the criteria for study-defined composite cardiac dysfunction. In fully adjusted models, higher z-lnSII and z-lnSIRI were independently associated with the endpoint (SII: OR 2.148, 95% CI 1.540–2.997; SIRI: OR 1.927, 95% CI 1.376–2.697; both *P* < 0.001). Associations remained significant in medication- and partial available-case oncology-adjusted sensitivity analyses and remained directionally consistent in *post hoc* sensitivity analyses using progressively higher follow-up BNP thresholds of 125, 150, 200, and 400 pg/mL. In five-fold logistic prediction analyses, the AUC increased from 0.633 for the base model to 0.745 with SII, 0.733 with SIRI, and 0.745 with both markers.

**Conclusion:**

Baseline SII and SIRI were independently associated with study-defined composite cardiac dysfunction during NSAI therapy and provided incremental discrimination beyond conventional clinical variables.

## 1 Introduction

Breast cancer is the most commonly diagnosed malignancy in women worldwide, with an estimated 2,296,840 new cases globally in 2022 (1). Owing to substantial advances in screening, diagnosis, and systemic treatment, survival outcomes for patients with breast cancer have improved markedly. As survival has increased, treatment-related long-term complications have become increasingly important determinants of prognosis in cancer survivors. Among these, cardiovascular toxicity has emerged as a major non-cancer cause of morbidity and mortality, making early recognition of cancer therapy-related cardiac injury an important challenge in cardio-oncology (2–5).

Among all molecular subtypes of breast cancer, approximately 70% are hormone receptor-positive (HR+), defined by estrogen receptor and/or progesterone receptor expression (6, 7). For postmenopausal women with HR + breast cancer, non-steroidal aromatase inhibitors (NSAIs), mainly letrozole and anastrozole, are widely used as standard adjuvant endocrine agents because they effectively reduce the risk of recurrence. NSAIs suppress aromatase activity and thereby inhibit peripheral estrogen biosynthesis, resulting in sustained estrogen deprivation (8). However, these drugs are commonly administered for prolonged periods, often 5–10 years, in an aging population already characterized by increasing baseline cardiovascular vulnerability. In this setting, even subtle treatment-related cardiac dysfunction may have important clinical consequences, particularly when it remains unrecognized until overt cardiovascular events or heart failure become clinically apparent (9, 10). Long-term clinical and population-based studies have linked aromatase inhibitor use to adverse cardiovascular outcomes, including heart failure, arrhythmia, and ischemic heart disease (11–13). These observations suggest that NSAI-related cardiac dysfunction may not primarily manifest as acute overt toxicity, but rather as a more insidious and progressive decline in cardiac function. Accordingly, cardiac functional and biomarker deterioration observed during NSAI therapy may represent an early phenotype warranting further investigation before overt cardiovascular events develop.

A key unresolved question is why only a subset of patients develop such dysfunction during NSAI therapy. This pattern suggests that drug exposure alone may be insufficient to explain risk and that baseline host susceptibility may also be important. Increasing evidence indicates that cancer therapy-related cardiovascular injury involves not only direct tissue damage but also broader immune-inflammatory processes, including chronic low-grade inflammation, innate immune activation, oxidative stress, endothelial dysfunction, and maladaptive remodeling (14). This framework may be especially relevant in postmenopausal women, in whom estrogen deprivation, cardiovascular aging, and inflammaging may converge to create a more vulnerable biological background (15, 16). Within this context, readily available blood-derived inflammatory indices may provide a practical means of capturing pre-treatment immune-inflammatory susceptibility. The systemic immune-inflammation index (SII), calculated from neutrophil, platelet, and lymphocyte counts, reflects the interplay among inflammatory activation, thrombo-inflammatory activity, and immune regulation. The systemic inflammation response index (SIRI), derived from neutrophil, monocyte, and lymphocyte counts, may further capture monocyte-related innate immune responses and inflammatory remodeling. Both indices have shown prognostic relevance in cardiovascular disease and cancer, yet their associations with study-defined composite cardiac dysfunction during NSAI treatment remain unknown (17–19).

Therefore, we investigated the associations of baseline SII and SIRI with study-defined composite cardiac dysfunction in postmenopausal women with HR + breast cancer receiving NSAIs. We further evaluated whether incorporation of these immune-inflammatory indices could improve risk discrimination beyond conventional clinical variables. We hypothesized that elevated baseline immune-inflammatory burden, as reflected by SII and SIRI, may identify patients at increased risk of developing study-defined composite cardiac dysfunction during NSAI therapy and may provide incremental value for early risk assessment and individualized cardiovascular surveillance.

## 2 Materials and methods

### 2.1 Study design and population

A single-center retrospective cohort study was conducted at Shanghai Renji Hospital, including consecutive postmenopausal women with pathologically confirmed HR + breast cancer who received NSAIs between January 2020 and April 2024. Standardized clinical, laboratory, and echocardiographic data obtained during routine care were retrospectively reviewed. Inclusion criteria were as follows: (1) postmenopausal women; (2) pathologically confirmed HR + breast cancer; (3) treatment with letrozole or anastrozole; and (4) receipt of NSAI therapy for at least 3 months; and (5) availability of baseline echocardiography before NSAI initiation and at least one post-NSAI follow-up echocardiographic or BNP assessment in the routine clinical record for endpoint ascertainment. Exclusion criteria were as follows: (1) interruption, discontinuation, or regimen change during NSAI therapy; (2) moderate or greater valvular heart disease, rheumatic heart disease, hypertrophic cardiomyopathy, infiltrative cardiomyopathy, restrictive cardiomyopathy, adult congenital heart disease, or other significant structural heart disease; (3) acute infection, active inflammatory disease, or other conditions likely to substantially affect baseline inflammatory indices at the time of blood sampling; and (4) an expected survival of <6 months or severe medical conditions precluding standardized follow-up assessment.

A total of 359 patients with hormone receptor-positive breast cancer treated with letrozole or anastrozole between January 2020 and April 2024 were screened. Of these, 90 were excluded, including 80 who did not meet the predefined treatment-duration or follow-up assessment window, 8 with treatment interruption, discontinuation, regimen change, or poor adherence, and 2 with baseline cardiac dysfunction. The final analytic cohort consisted of 269 patients (Figure 1).

**Figure 1:**
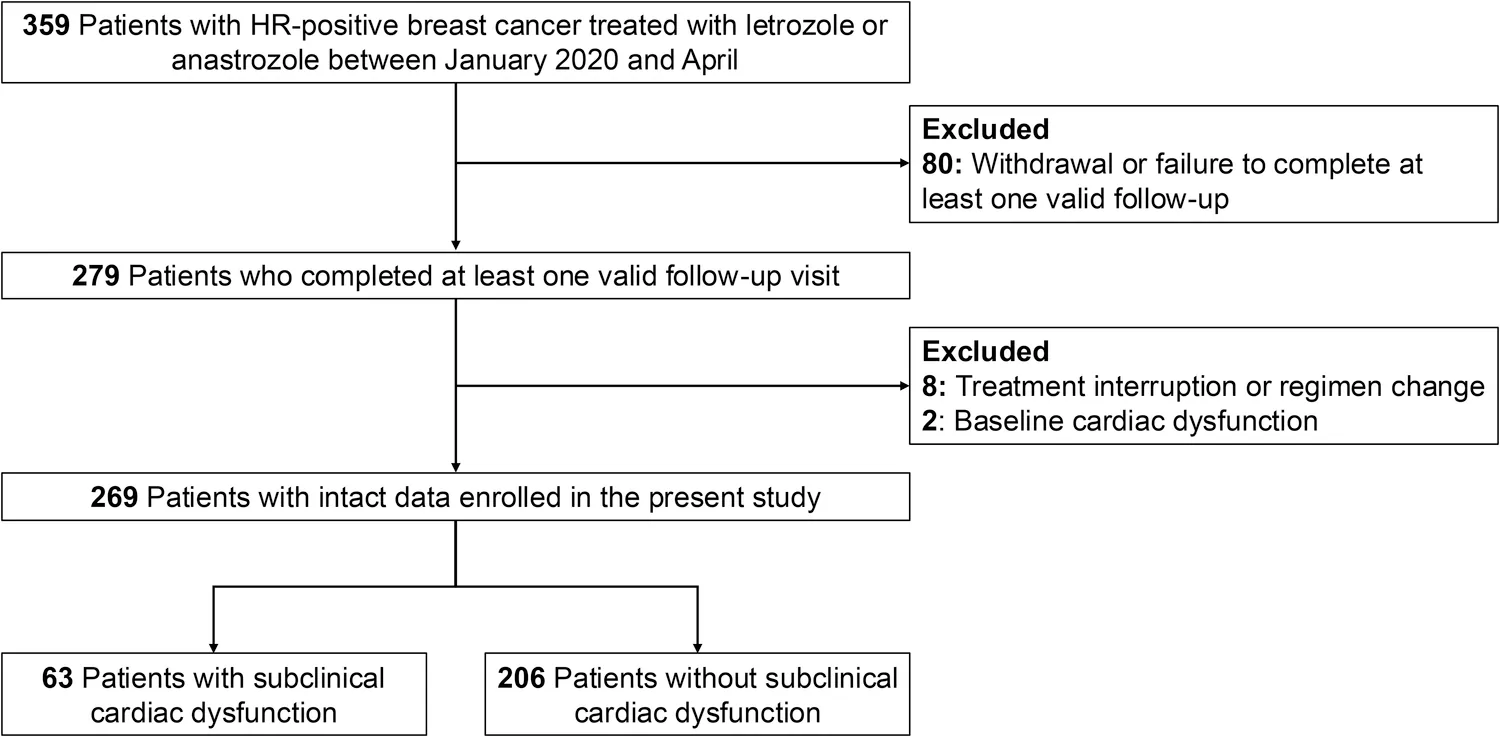
Flowchart of study population selection. Flow diagram showing patient screening, exclusions, and inclusion in the final analytic cohort. NSAI, non-steroidal aromatase inhibitor.

The study protocol was approved by the Ethics Committee of Renji Hospital, Shanghai Jiao Tong University School of Medicine (Approval No. KY2020-189-CR-02). The study was conducted in accordance with the Declaration of Helsinki and relevant institutional requirements.

### 2.2 Data collection and outcome definition

Electronic medical records from Shanghai Renji Hospital were retrospectively reviewed to obtain demographic, clinical, oncologic, laboratory, and echocardiographic data. Demographic and treatment-related variables included age at NSAI initiation, body mass index (BMI), NSAI type (letrozole or anastrozole), and observation duration. Observation duration was defined as the interval from NSAI initiation to the last available post-NSAI echocardiographic or BNP assessment used for endpoint ascertainment. Medical history, including hypertension, diabetes mellitus, hyperlipidemia, and prior cardiovascular disease, as well as baseline cardiovascular medication use, was also recorded.

Available breast cancer-related characteristics and oncologic treatments included tumor laterality, histologic subtype and grade, TNM stage, molecular subtype, surgery type, HER2 status and HER2-targeted therapy, radiotherapy, and CDK4/6 inhibitor use. These variables were summarized descriptively, and a clinically selected subset with sufficient data completeness was included in the available-case oncology-adjusted sensitivity analyses.

Baseline laboratory variables were obtained from routine hematologic and biochemical assessments. Neutrophil, lymphocyte, monocyte, and platelet counts were collected and used to calculate SII and SIRI. Serum creatinine was retrieved, and estimated glomerular filtration rate (eGFR) was obtained from the clinical record or recalculated from available source measurements where necessary. Baseline and follow-up BNP concentrations were obtained from routine clinical laboratory records. All measurements were performed in the clinical laboratory of Renji Hospital using standard laboratory procedures.

Baseline LVEF and BNP values were defined using eligible measurements obtained before NSAI initiation. For each endpoint component, the follow-up value was defined as the last available post-NSAI measurement of the corresponding marker in the routine clinical record. The primary endpoint was study-defined composite cardiac dysfunction, operationally defined in this study as either: (1) an absolute decline in LVEF of ≥10 percentage points from baseline; or (2) a relative increase in BNP of ≥40% from baseline together with a follow-up BNP concentration ≥100 pg/mL. Patients meeting either criterion were classified as having study-defined composite cardiac dysfunction. The BNP-based component required both a relative increase of ≥40% from baseline and a follow-up BNP concentration of ≥100 pg/mL. The absolute threshold was intended to reduce classification driven solely by a large proportional increase from a very low baseline value and was not considered a population-specific, age-adjusted, or externally validated cardio-oncology cutoff.

### 2.3 Echocardiographic assessment

Echocardiographic assessment was performed using two-dimensional transthoracic echocardiography with an E9 color Doppler ultrasound system (GE Healthcare, USA) equipped with a 1.8–3.6 MHz transducer. Patients were examined in the left lateral decubitus position. Standard parasternal long-axis, apical four-chamber, and apical two-chamber views were obtained according to routine clinical echocardiographic protocols. In the parasternal long-axis view, aortic root diameter, left ventricular end-diastolic diameter, interventricular septal thickness, and left ventricular posterior wall thickness were measured at end-diastole, whereas left atrial diameter and left ventricular end-systolic diameter were measured at end-systole. LVEF was measured from the apical four-chamber and two-chamber views using the biplane Simpson method. For this retrospective study, baseline and follow-up LVEF values were extracted from routine clinical echocardiographic reports. Global longitudinal strain was not routinely available during the study period and therefore could not be incorporated into the endpoint definition.

### 2.4 Formulas used to calculate the SII and SIRI index

SII = platelet count × neutrophil count/lymphocyte count.

SIRI = neutrophil count ×  monocyte count/lymphocyte count.

### 2.5 Statistical analysis

All analyses were performed using R version 4.4.2 and Python version 3.11.8. All tests were two-sided, with *P* < 0.05 considered statistically significant. Before analysis, variable coding, ranges, distributions, and consistency across clinical, laboratory, and echocardiographic records were examined.

Variable-specific missingness is reported in Supplementary Table S1. Values recoverable from source records or deterministically derivable from available measurements were completed before statistical imputation. For the primary association analyses, remaining missing covariate data were handled using multiple imputation by chained equations under the missing-at-random assumption. The imputation model included the study outcome, all variables in the primary regression models, the component variables of SII and SIRI, and prespecified auxiliary variables, including BMI, systolic and diastolic blood pressure, serum creatinine, LDL-C, baseline BNP, left ventricular fractional shortening, and left ventricular end-diastolic and end-systolic diameters. Continuous variables were imputed using predictive mean matching and binary variables using logistic regression. Fifty imputed datasets were generated, with 20 iterations per imputation. SII and SIRI were recalculated within each imputed dataset and subsequently natural log-transformed and standardized. The outcome was included in the imputation model but was not imputed. Regression estimates were pooled according to Rubin's rules. Convergence was assessed using trace plots and comparisons of observed and imputed distributions.

Continuous variables were assessed for normality using the Shapiro–Wilk test. Normally distributed variables are presented as mean ± standard deviation and were compared using the independent-samples t test; non-normally distributed variables are presented as median (interquartile range) and were compared using the Mann–Whitney U test. Categorical variables are presented as number (percentage) and were compared using the *χ*^2^ test or Fisher's exact test, as appropriate.

Associations of baseline SII and SIRI with study-defined composite cardiac dysfunction were evaluated using logistic regression. Model 1 was unadjusted. Model 2 was adjusted for age, NSAI type, and observation duration. Model 3 was additionally adjusted for hypertension, diabetes mellitus, history of cardiovascular disease, baseline LVEF, and eGFR. *Post hoc* component-attribution analyses were conducted using the corresponding paired-measurement populations. An event was attributed to the LVEF component when the patient met the original composite endpoint and follow-up LVEF was lower than baseline, and to the BNP component when the patient met the original composite endpoint and follow-up BNP was higher than baseline. The two analyses were conducted separately, overlap was allowed, and all other component-evaluable patients served as the comparison group. Fully adjusted analyses included the same covariates as Model 3. These directional attribution analyses were not used to redefine the primary endpoint or to reconstruct mutually exclusive endpoint components. Covariates were prespecified according to clinical relevance and prior evidence. Multicollinearity was assessed using variance inflation factors. Results are reported as odds ratios with 95% confidence intervals. SII and SIRI were also analyzed as tertiles, with the lowest tertile as the reference category. Restricted cubic spline models were used to assess potential nonlinear associations, with the cohort median values of SII (396) and SIRI (0.66) used as the respective reference values.

Prediction models were constructed using a base clinical feature set comprising age, NSAI type, observation duration, hypertension, diabetes mellitus, history of cardiovascular disease, baseline LVEF, and eGFR, with subsequent addition of SII, SIRI, or both indices. Model performance was evaluated using stratified five-fold out-of-fold predictions. Missing-value handling and scaling were nested within each resampling iteration, with all preprocessing parameters estimated exclusively from the training fold and subsequently applied to the held-out validation fold. Discrimination was assessed using AUCs with 95% CIs, and correlated AUCs were compared using DeLong tests. Calibration was evaluated using calibration plots, calibration intercepts, calibration slopes, and Brier scores. Decision-curve analysis was used to assess net benefit, whereas category-free NRI and IDI were calculated as exploratory measures of incremental reclassification.

Several sensitivity analyses were performed to assess the robustness of the primary associations. To examine whether the findings depended on the selected absolute BNP threshold, *post hoc* sensitivity analyses were conducted using follow-up BNP thresholds of 100, 125, 150, 200, and 400 pg/mL. The original 100-pg/mL analysis retained all primary events. At higher thresholds, events classified exclusively through the BNP branch were retained only when the observed follow-up BNP met the specified cutoff, whereas BNP-threshold-independent events were unchanged. Models 1–3 were refitted for each threshold. Because no validated age-adjusted BNP criterion was available for this specific cardio-oncology outcome, age and eGFR were included as covariates in Model 3 rather than deriving data-dependent age-specific cutoffs. The influence of extreme SII and SIRI values was further evaluated using Winsorization and trimming at the 1st–99th and 2.5th–97.5th percentiles. A medication-adjusted model additionally included baseline beta-blocker, ACEI/ARB/ARNI, and statin use and was restricted to patients with complete cardiovascular medication records. A partial available-case oncology-adjusted sensitivity model additionally incorporated left-sided breast cancer, invasive ductal carcinoma, TNM stage II-IV, Luminal B subtype, HER2 IHC 3+, HER2-targeted therapy, radiotherapy, CDK4/6 inhibitor use, and mastectomy or radical surgery. Complete data for all nine oncology covariates were required; patients with missing data for any of these variables were excluded from this analysis. Characteristics of patients included in and excluded from the complete-case analysis were compared and are reported in Supplementary Table S3. Subgroup analyses were conducted according to age, hypertension status, and renal function, with interaction terms used to assess effect modification.

Exploratory machine-learning analyses were conducted using four prespecified feature sets: the base clinical feature set, base + SII, base + SIRI, and base + both indices. Random forest was used as the primary exploratory algorithm, whereas extra trees, gradient boosting, AdaBoost, and Gaussian naive Bayes classifiers were evaluated as complementary models. The candidate feature sets and model hyperparameters were prespecified, and no feature screening or hyperparameter search was performed using the full dataset. Within each cross-validation iteration, missing-value imputation, scaling where applicable, class-imbalance handling, model fitting, and any data-driven feature selection were performed exclusively using the training data; held-out folds were used only for performance evaluation. Primary performance estimates were obtained using stratified five-fold out-of-fold predictions. As a *post hoc* robustness analysis, nested cross-validation with five outer and three inner folds was additionally performed. The outer folds were used exclusively for evaluation, whereas all preprocessing and model-development procedures were restricted to the corresponding training data. Given the modest sample size and event count, all machine-learning analyses were considered exploratory and potentially optimistic.

## 3 Results

### 3.1 Baseline characteristics

As shown in Table 1, 269 postmenopausal women with HR + breast cancer receiving NSAIs were included, of whom 63 (23.4%) met the criteria for study-defined composite cardiac dysfunction. Compared with patients without the endpoint, those with study-defined composite cardiac dysfunction were older, had a higher prevalence of cardiovascular comorbidities, and exhibited higher baseline SII and SIRI levels. The distribution of NSAI type and observation duration did not differ significantly between groups. The median interval from NSAI initiation to the final follow-up assessment was 334 days and was comparable between groups. Baseline ACEI/ARB/ARNI use was more frequent among patients with the endpoint, whereas the use of other cardiovascular medications did not differ significantly. Available oncologic characteristics and treatment exposures were generally balanced between groups, and a clinically selected subset was included in the oncology-adjusted sensitivity analysis.

**Table 1:** Baseline characteristics of the study population.

Variable	Overall cohort	Without cardiac dysfunction	With cardiac dysfunction	*P* value
Demographic and clinical				
Age, years	64.6 ± 9.0	64.0 ± 9.3	66.5 ± 7.5	0.032
BMI, kg/m^2^	24.0 ± 3.4	24.1 ± 3.4	23.7 ± 3.7	0.480
Systolic blood pressure, mmHg	130.4 ± 15.2	130.1 ± 15.3	131.5 ± 14.9	0.498
Diastolic blood pressure, mmHg	77.1 ± 9.6	77.4 ± 10.3	76.1 ± 6.8	0.255
Cardiovascular risk				
Hypertension	75/269 (27.9%)	49/206 (23.8%)	26/63 (41.3%)	0.007
Diabetes mellitus	34/269 (12.6%)	21/206 (10.2%)	13/63 (20.6%)	0.029
Hyperlipidemia	19/269 (7.1%)	14/206 (6.8%)	5/63 (7.9%)	0.780
History of cardiovascular disease	16/269 (5.9%)	7/206 (3.4%)	9/63 (14.3%)	0.004
Breast cancer and oncologic treatment				
Left-sided breast cancer	126/221 (57.0%)	92/164 (56.1%)	34/57 (59.6%)	0.756
Invasive ductal carcinoma	175/221 (79.2%)	131/164 (79.9%)	44/57 (77.2%)	0.706
Histological grade 2–3	175/193 (90.7%)	132/145 (91.0%)	43/48 (89.6%)	0.777
TNM stage II-IV	99/205 (48.3%)	73/154 (47.4%)	26/51 (51.0%)	0.747
Luminal B subtype	99/215 (46.0%)	78/160 (48.8%)	21/55 (38.2%)	0.210
HER2 IHC score	1.00 (0.00, 2.00)	1.00 (1.00, 2.00)	1.00 (0.00, 3.00)	0.610
HER2 IHC 3+	52/215 (24.2%)	36/159 (22.6%)	16/56 (28.6%)	0.371
HER2-targeted therapy	44/219 (20.1%)	31/162 (19.1%)	13/57 (22.8%)	0.567
Pyrotinib	10/219 (4.6%)	7/162 (4.3%)	3/57 (5.3%)	0.723
Trastuzumab	34/219 (15.5%)	23/162 (14.2%)	11/57 (19.3%)	0.397
Pertuzumab	20/219 (9.1%)	13/162 (8.0%)	7/57 (12.3%)	0.422
Radiotherapy	14/219 (6.4%)	9/162 (5.6%)	5/57 (8.8%)	0.365
CDK4/6 inhibitor	22/219 (10.0%)	19/162 (11.7%)	3/57 (5.3%)	0.206
Mastectomy/radical surgery	198/215 (92.1%)	149/161 (92.5%)	49/54 (90.7%)	0.771
Breast-conserving surgery	14/215 (6.5%)	10/161 (6.2%)	4/54 (7.4%)	0.754
Baseline cardiac and laboratory				
Baseline LVEF, %	66.4 ± 4.5	66.0 ± 4.0	67.6 ± 5.6	0.041
Baseline BNP, pg/mL	24.5 (15.8, 46.0)	27.0 (16.0, 45.5)	22.0 (15.0, 47.0)	0.468
LV fractional shortening, %	36 (34, 39)	36 (34, 38)	38 (35.8, 41)	0.002
Aortic root diameter, mm	30 (28, 32)	30 (28, 32)	29 (28, 32)	0.281
Left atrial diameter, mm	35 (32, 38)	35 (32, 38)	35 (33, 37)	0.910
Interventricular septal thickness, mm	8 (7.6, 9)	8 (7.6, 9)	8.1 (8, 9)	0.370
LV end-diastolic diameter, mm	44.80 ± 4.21	44.95 ± 4.04	44.31 ± 4.74	0.340
LV end-systolic diameter, mm	28 (26, 30)	29 (27, 31)	27 (25, 30)	0.01
eGFR, mL/min/1.73 m^2^	94.0 (88.0, 101.0)	96.0 (89.0, 101.0)	92.0 (86.0, 95.5)	0.027
LDL-C, mmol/L	2.91 (2.29, 3.39)	3.02 (2.45, 3.40)	2.62 (2.18, 3.15)	0.025
Hematologic and inflammatory markers				
Neutrophil count, × 10^9^/L	3.07 (2.14, 4.13)	2.79 (2.01, 3.73)	4.07 (2.98, 5.34)	<0.001
Lymphocyte count, × 10^9^/L	1.67 (1.27, 2.12)	1.73 (1.35, 2.16)	1.47 (1.09, 1.98)	0.015
Monocyte count, × 10^9^/L	0.38 (0.30, 0.50)	0.37 (0.29, 0.49)	0.43 (0.35, 0.52)	0.053
NLR	1.66 (1.27, 2.68)	1.51 (1.24, 2.31)	2.68 (1.87, 4.33)	<0.001
MLR	0.22 (0.17, 0.30)	0.21 (0.17, 0.27)	0.27 (0.20, 0.44)	0.002
SII	396.2 (270.7, 645.9)	352.9 (261.0, 512.5)	663.9 (399.3, 1,072.1)	<0.001
SIRI	0.7 (0.4, 1.2)	0.6 (0.4, 0.9)	1.1 (0.7, 1.9)	<0.001
NSAI treatment				
Letrozole	219/269 (81.4%)	166/206 (80.6%)	53/63 (84.1%)	0.527
Anastrozole	50/269 (18.6%)	40/206 (19.4%)	10/63 (15.9%)	0.527
Observation duration, days	334.0 (179.0, 562.0)	318.5 (169.0, 555.8)	409.0 (211.0, 576.0)	0.134
Baseline cardiovascular medications				
Calcium-channel blocker	33/245 (13.5%)	21/182 (11.5%)	12/63 (19.0%)	0.132
ACEI/ARB/ARNI	25/245 (10.2%)	13/182 (7.1%)	12/63 (19.0%)	0.007
Beta-blocker	19/245 (7.8%)	11/182 (6.0%)	8/63 (12.7%)	0.103
Statin	17/245 (6.9%)	11/182 (6.0%)	6/63 (9.5%)	0.390
Diuretic	6/244 (2.5%)	4/181 (2.2%)	2/63 (3.2%)	0.650

Data are presented as mean ± SD, median (IQR), or *n* (%). BNP, B-type natriuretic peptide; eGFR, estimated glomerular filtration rate; LVEF, left ventricular ejection fraction; MLR, monocyte-to-lymphocyte ratio; NLR, neutrophil-to-lymphocyte ratio; PLR, platelet-to-lymphocyte ratio; SII, systemic immune-inflammation index; SIRI, systemic inflammation response index.

An available-case comparison between included and excluded patients is presented in Supplementary Table S2. No statistically significant differences were observed in several available laboratory and echocardiographic variables. However, cardiovascular risk factors, NSAI type, medication use, and several oncologic and clinical variables were unavailable for most excluded patients. Therefore, these restricted comparisons do not establish cohort comparability, and substantial residual selection bias cannot be excluded.

### 3.2 Baseline SII and SIRI were independently associated with study-defined composite cardiac dysfunction

Logistic regression analyses were performed to evaluate the associations of baseline SII and SIRI with study-defined composite cardiac dysfunction after natural-log transformation and z standardization. Both indices were associated with higher odds of the endpoint across the crude, partially adjusted, and fully adjusted models (Figure 2).

**Figure 2:**
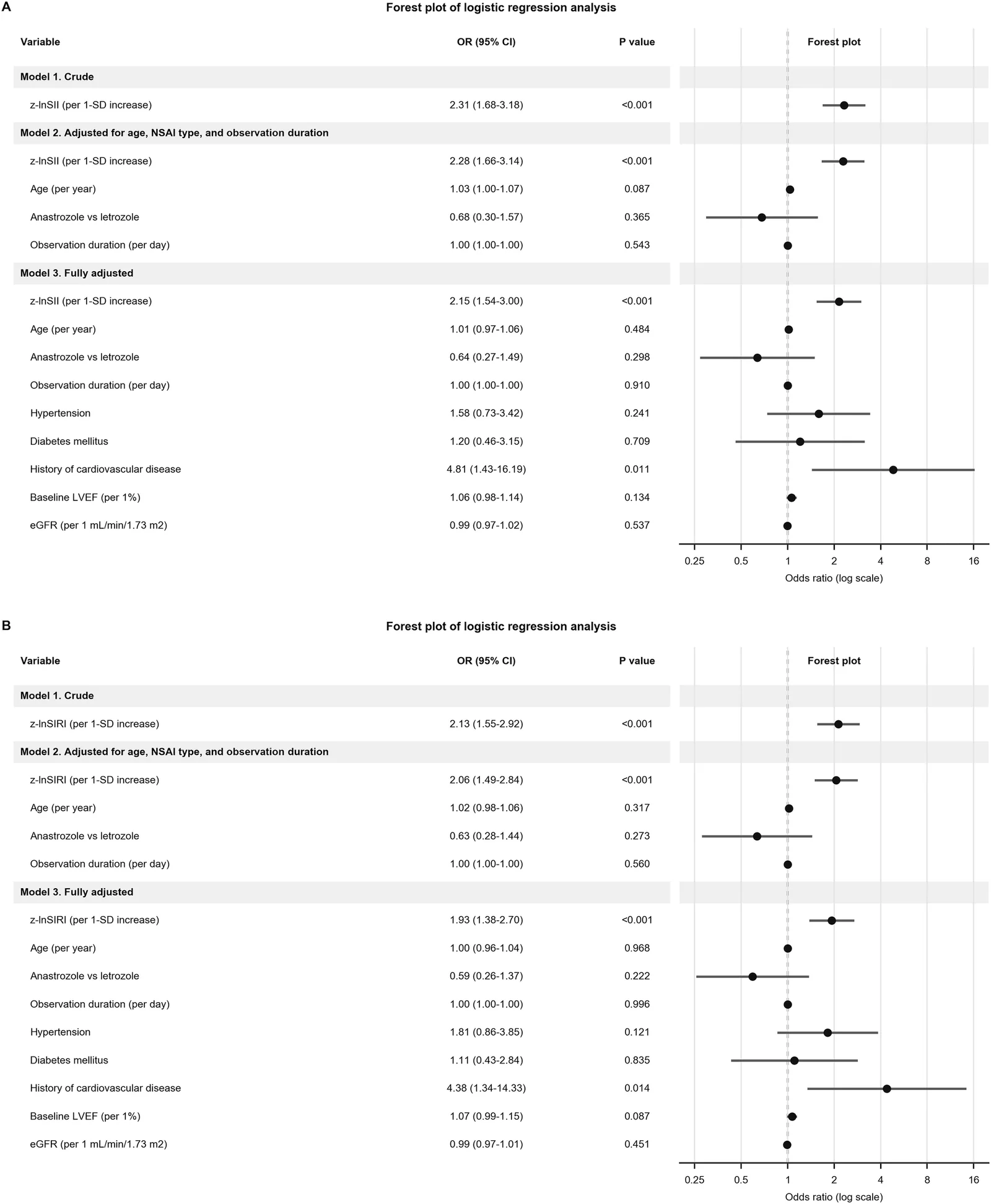
Associations of baseline SII and SIRI with subclinical cardiac dysfunction in logistic regression analyses. **(A)** Association of z-standardized natural log-transformed SII with the endpoint. **(B)** Association of z-standardized natural log-transformed SIRI with the endpoint. Forest plots show ORs and 95% CIs from the unadjusted, partially adjusted, and fully adjusted models. SII, systemic immune-inflammation index; SIRI, systemic inflammation response index; OR, odds ratio; CI, confidence interval.

For each 1-SD increase in z-lnSII, the ORs were 2.31 (95% CI, 1.68–3.18) in the crude model, 2.28 (95% CI, 1.66–3.14) in the partially adjusted model, and 2.15 (95% CI, 1.54–3.00) in the fully adjusted model (all *P* < 0.001). The corresponding ORs for z-lnSIRI were 2.13 (95% CI, 1.55–2.92), 2.06 (95% CI, 1.49–2.84), and 1.93 (95% CI, 1.38–2.70), respectively (all *P* < 0.001).

### 3.3 *Post hoc* BNP-threshold sensitivity and component-attribution analyses

In *post hoc* BNP-threshold sensitivity analyses, the 100-pg/mL reference analysis retained 63 events. At follow-up BNP thresholds of 125, 150, 200, and 400 pg/mL, 47, 47, 46, and 43 events remained, respectively. Fully adjusted ORs for z-lnSII were 2.15, 2.84, 2.84, 3.03, and 2.77, whereas those for z-lnSIRI were 1.93, 2.49, 2.49, 2.60, and 2.29, respectively (all *P* < 0.001; Supplementary Figure S1).

Among the 63 primary events, nine initially met both attribution rules. These overlapping cases were subsequently classified using a support vector machine (SVM), resulting in 33 events attributed to an LVEF decline and 30 to a BNP rise. In the fully adjusted analyses, z-lnSII and z-lnSIRI were associated with the LVEF-attributed component among 241 patients (SII: OR 2.09, 95% CI 1.36–3.20, *P* = 0.001; SIRI: OR 1.73, 95% CI 1.15–2.61, *P* = 0.009), whereas the associations with the BNP-attributed component among 88 patients were weaker and nonsignificant (SII: OR 1.46, 95% CI 0.88–2.40, *P* = 0.143; SIRI: OR 1.38, 95% CI 0.86–2.21, *P* = 0.180).

### 3.4 Excess risk was concentrated in the highest tertiles of SII and SIRI

Tertile-based analyses showed that the increased risk of subclinical cardiac dysfunction was concentrated in the highest tertile of both SII and SIRI, whereas the middle tertile was not significantly associated with the outcome (Figure 3).

**Figure 3:**
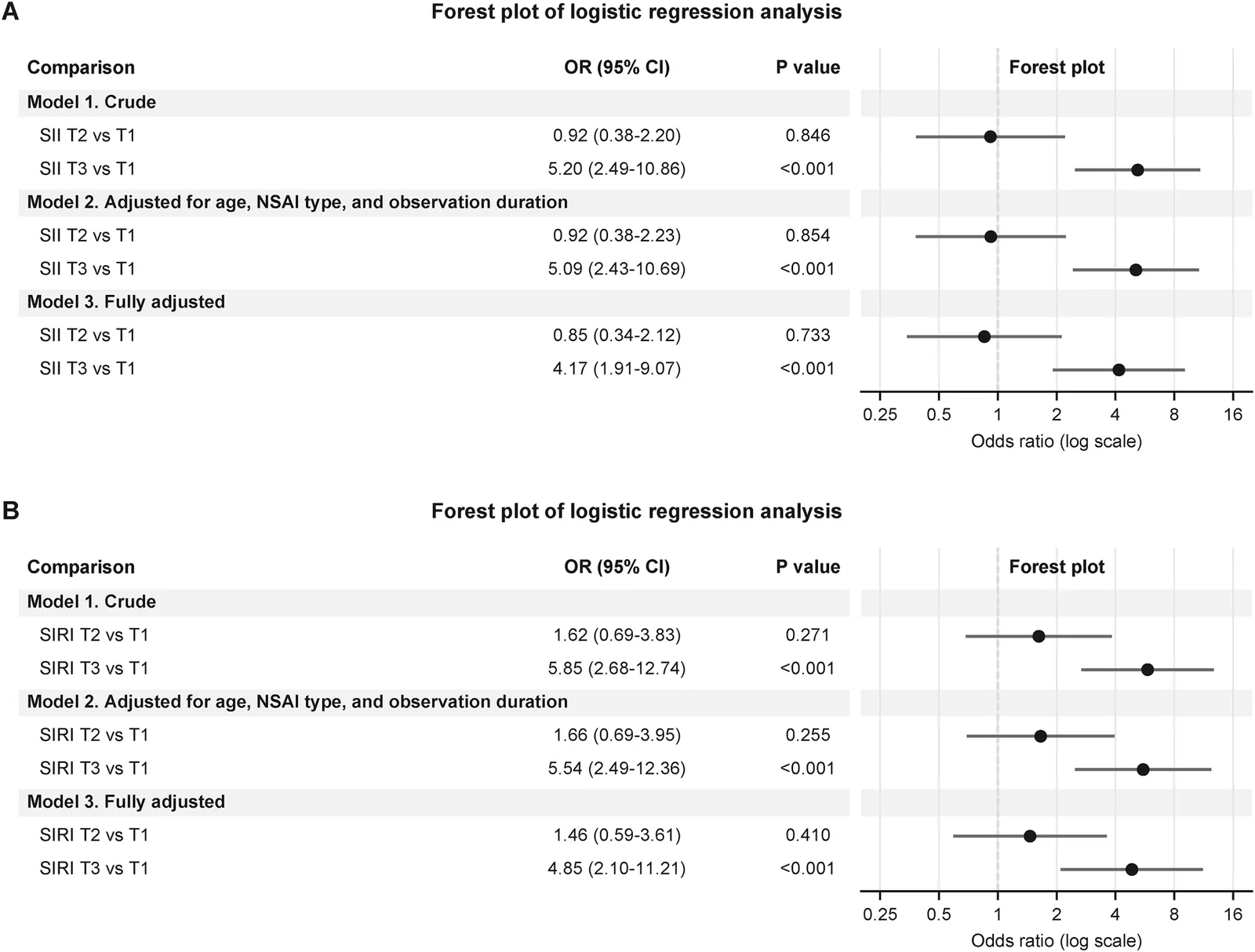
Tertile-based associations of baseline SII and SIRI with study-defined composite cardiac dysfunction. **(A)** Associations across SII tertiles. **(B)** Associations across SIRI tertiles. The lowest tertile was used as the reference category. Forest plots show ORs and 95% CIs from the unadjusted, partially adjusted, and fully adjusted models. SII, systemic immune-inflammation index; SIRI, systemic inflammation response index; OR, odds ratio; CI, confidence interval.

For SII, compared with the lowest tertile, the highest tertile was consistently associated with a markedly increased risk of cardiac functional deterioration across all models, including the fully adjusted model (OR 4.17, 95% CI 1.91–9.07, *P* < 0.001). By contrast, the middle tertile was not significantly associated with the endpoint in either crude or adjusted analyses. A similar pattern was observed for SIRI. Relative to the lowest tertile, the highest tertile remained strongly associated with cardiac functional deterioration after full adjustment (OR 4.85, 95% CI 2.10–11.21, *P* < 0.001), whereas the middle tertile showed no statistically significant association with risk. These findings suggest that excess risk was mainly enriched among patients with high baseline immune-inflammatory burden.

### 3.5 SII and SIRI showed nonlinear associations with study-defined composite cardiac dysfunction

Restricted cubic spline analyses further demonstrated significant nonlinear associations of both baseline SII and SIRI with the risk of study-defined composite cardiac dysfunction (Figure 4). For SII, the overall association was significant (P overall < 0.001), and the test for non-linearity was also significant (*P* for non-linearity = 0.0027). Using the cohort median SII value of 396 as the reference, the estimated risk remained relatively stable at lower levels but increased progressively at higher SII values. A similar pattern was observed for SIRI. The overall association between SIRI and study-defined composite cardiac dysfunction was significant (*P* overall < 0.001), with evidence of non-linearity (*P* for non-linearity = 0.0072). Using the cohort median SIRI value of 0.66 as the reference, the estimated odds of study-defined composite cardiac dysfunction increased more steeply above this level and then gradually plateaued at higher values.

**Figure 4:**
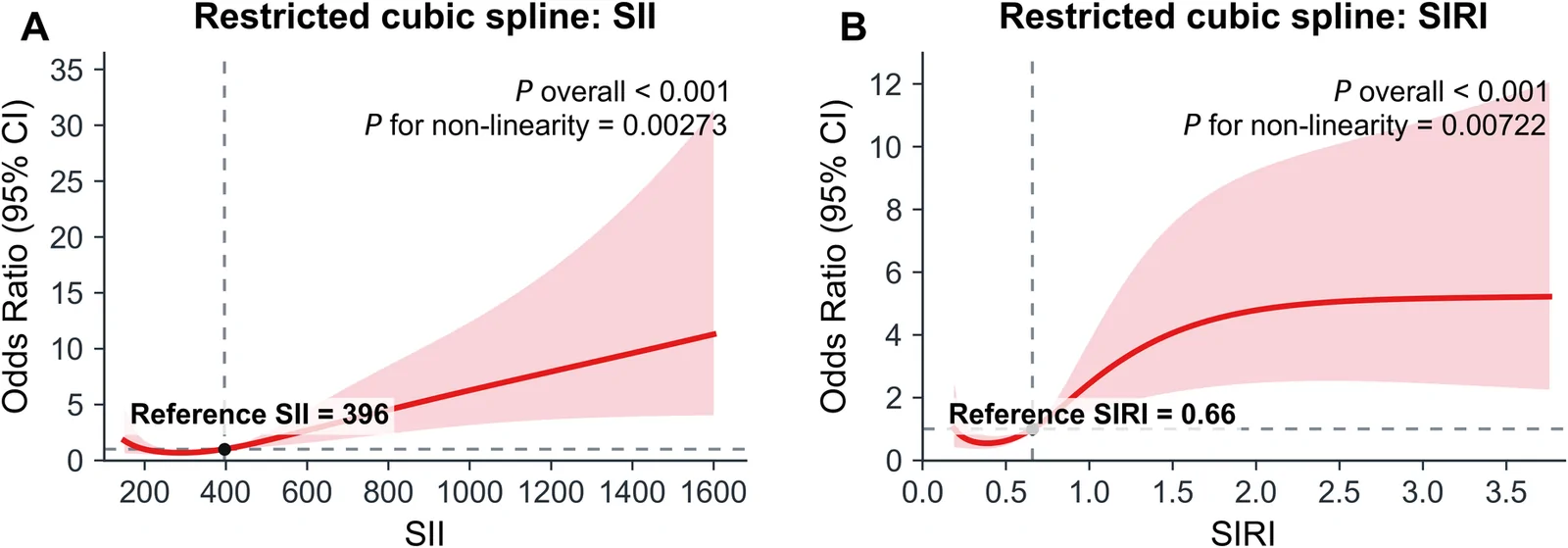
Nonlinear associations of baseline SII and SIRI with study-defined composite cardiac dysfunction. **(A)** Restricted cubic spline analysis of SII. **(B)** Restricted cubic spline analysis of SIRI. Solid lines represent adjusted ORs, and shaded areas represent 95% CIs. The cohort median values of SII (396) and SIRI (0.66) were used as the respective reference values. SII, systemic immune-inflammation index; SIRI, systemic inflammation response index; OR, odds ratio; CI, confidence interval.

### 3.6 Baseline SII and SIRI improved discrimination beyond conventional clinical variables

Using stratified five-fold out-of-fold logistic predictions with fold-wise preprocessing, the base clinical model had an AUC of 0.633 (95% CI 0.558–0.713). AUCs were 0.745 (95% CI 0.663–0.818) after adding SII, 0.733 (95% CI 0.659–0.801) after adding SIRI, and 0.745 (95% CI 0.664–0.818) after adding both markers. Compared with the base model, AUC differences were 0.113 for SII, 0.100 for SIRI, and 0.112 for both markers; DeLong Z statistics were 3.453, 3.548, and 3.438, respectively; all *P* values were <0.001. The gain from adding both markers beyond SII alone was minimal (Figure 5).

**Figure 5:**
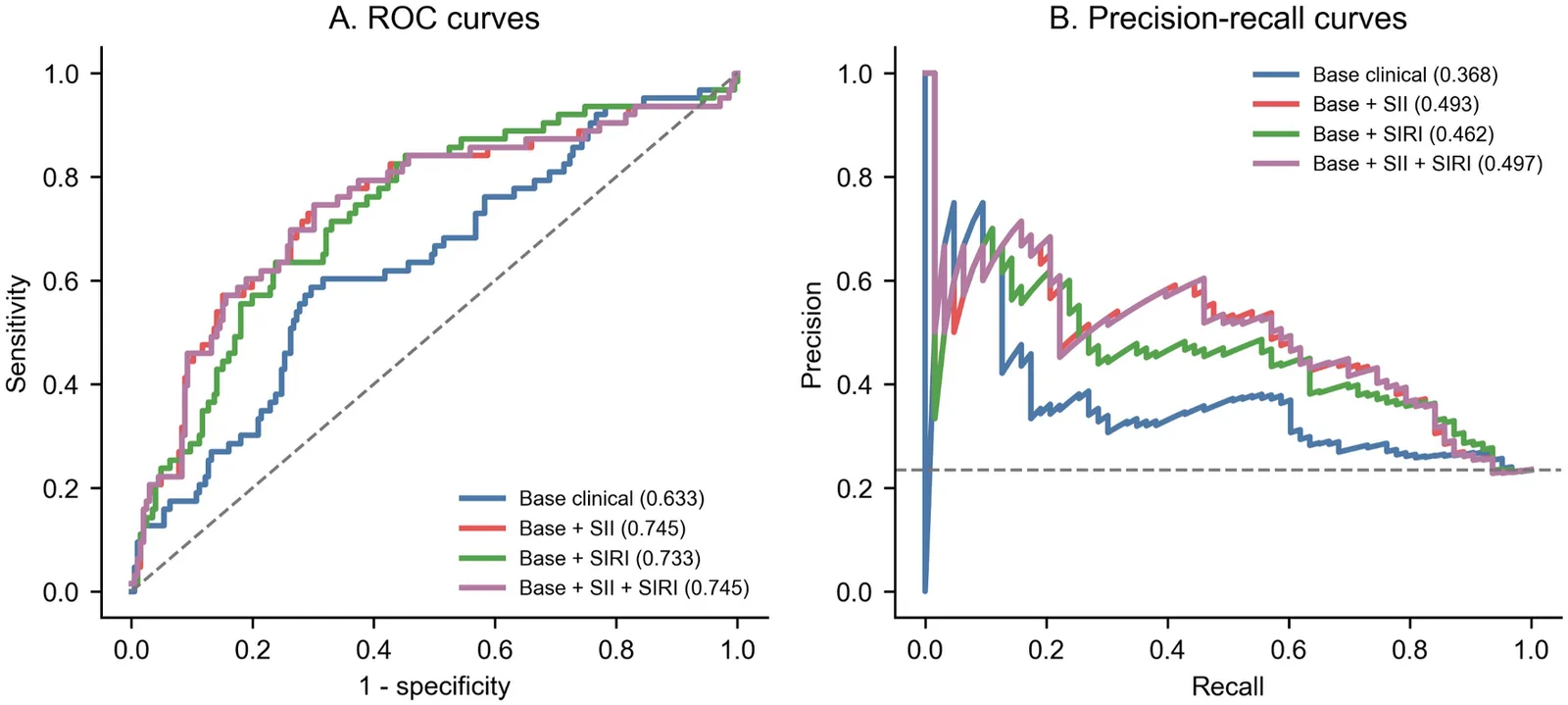
Internally validated discrimination of logistic prediction models for study-defined composite cardiac dysfunction. **(A)** Receiver operating characteristic curves. **(B)** Precision-recall curves. Models included the base clinical feature set alone or with the addition of SII, SIRI, or both indices. Predictions were generated using stratified five-fold cross-validation with fold-wise preprocessing. ROC, receiver operating characteristic; AUC, area under the receiver operating characteristic curve; SII, systemic immune-inflammation index; SIRI, systemic inflammation response index.

### 3.7 Calibration, decision-curve, and exploratory reclassification analyses

Calibration improved after adding inflammatory indices. The base model had a calibration intercept of −0.412, slope of 0.619, and Brier score of 0.173. Corresponding values were −0.203, 0.788, and 0.153 for base + SII; −0.232, 0.762, and 0.158 for base + SIRI; and −0.206, 0.788, and 0.153 for the combined model. Calibration plots nevertheless showed residual deviation from ideal agreement. Decision-curve findings were exploratory and varied across threshold probabilities (Figure 6).

**Figure 6:**
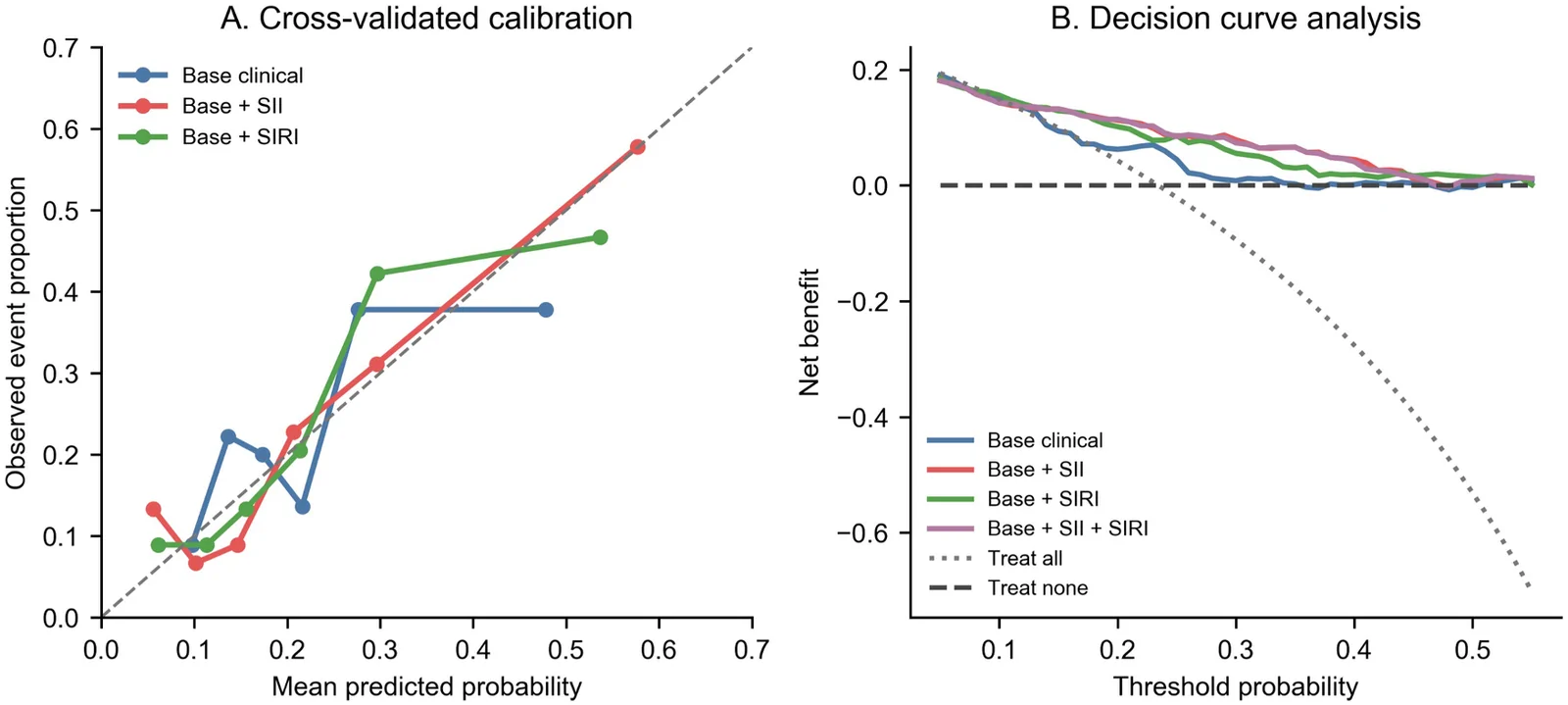
Cross-validated calibration and decision-curve analysis of logistic prediction models. **(A)** Quantile-based calibration plots for the base clinical model and models incorporating SII, SIRI, or both indices. The diagonal line represents ideal calibration. **(B)** Decision curves showing the net benefit of the four models across a range of threshold probabilities. SII, systemic immune-inflammation index; SIRI, systemic inflammation response index.

Exploratory category-free reclassification analyses favored the augmented models (base + SII: NRI 0.680, 95% CI 0.409–0.958; IDI 0.099, 95% CI 0.054–0.141; base + SIRI: NRI 0.597, 95% CI 0.327–0.863; IDI 0.069, 95% CI 0.034–0.106; all bootstrap *P* < 0.001). Because no prespecified clinical risk categories were available and the cohort was modest, these measures were reported as exploratory reclassification summaries.

### 3.8 Sensitivity analyses confirmed the robustness of the associations

Sensitivity analyses are summarized in Table 2. After Winsorization and trimming at the P1–P99 and P2.5–P97.5 levels, the associations of both indices with the endpoint remained statistically significant. The adjusted ORs for SII ranged from 2.185 to 2.431, whereas those for SIRI ranged from 2.026 to 2.673, with all *P* values <0.001. In the available-case oncology-adjusted model comprising 199 patients and 50 endpoint events, baseline SII and SIRI remained associated with study-defined cardiac functional deterioration (SII: OR per 1-SD increase, 2.410; 95% CI, 1.606–3.618; SIRI: OR per 1-SD increase, 2.129; 95% CI, 1.408–3.219; both *P* < 0.001). Seventy patients, including 13 endpoint events, were excluded from this analysis because of missing data for at least one required oncology covariate. Among these patients, 48 lacked all nine oncology variables, 12 lacked TNM stage only, and 10 had other combinations of missing oncology data. The comparisons between included and excluded patients are presented in Supplementary Table S3. In the medication-adjusted analysis comprising 245 patients and 62 endpoint events, further adjustment for baseline beta-blocker, ACEI/ARB/ARNI, and statin use yielded ORs of 2.159 (95% CI, 1.530–3.046; *P* < 0.001) for SII and 1.877 (95% CI, 1.329–2.652; *P* < 0.001) for SIRI.

**Table 2:** Sensitivity analyses of the associations of baseline SII and SIRI with study-defined composite cardiac dysfunction.

Biomarker	Analysis	*N*	Events, *n*	Adjusted OR per 1-SD increase (95% CI)	*P* value
SII	Primary analysis	269	63	2.148 (1.540, 2.997)	<0.001
	Winsorized (P1-P99)	269	63	2.185 (1.560, 3.060)	<0.001
	Trimmed (P1-P99)	263	60	2.328 (1.618, 3.350)	<0.001
	Winsorized (P2.5-P97.5)	269	63	2.254 (1.595, 3.186)	<0.001
	Trimmed (P2.5-P97.5)	255	56	2.431 (1.625, 3.637)	<0.001
	Oncology-adjusted	199	50	2.410 (1.606, 3.618)	<0.001
	Medication-adjusted	245	62	2.159 (1.530, 3.046)	<0.001
SIRI	Primary analysis	269	63	1.927 (1.376, 2.697)	<0.001
	Winsorized (P1-P99)	269	63	2.026 (1.436, 2.858)	<0.001
	Trimmed (P1-P99)	263	60	2.673 (1.784, 4.004)	<0.001
	Winsorized (P2.5-P97.5)	269	63	2.142 (1.495, 3.070)	<0.001
	Trimmed (P2.5-P97.5)	255	56	2.380 (1.547, 3.663)	<0.001
	Oncology-adjusted	199	50	2.129 (1.408, 3.219)	<0.001
	Medication-adjusted	245	62	1.877 (1.329, 2.652)	<0.001

ORs are reported per 1-SD increase in natural log-transformed SII or SIRI. The primary model was adjusted for age, NSAI type, observation duration, hypertension, diabetes mellitus, history of cardiovascular disease, baseline LVEF, and eGFR. The oncology-adjusted model additionally included left-sided breast cancer, invasive ductal carcinoma, TNM stage II-IV, Luminal B subtype, HER2 IHC 3+, HER2-targeted therapy, radiotherapy, CDK4/6 inhibitor use, and mastectomy or radical surgery and was restricted to patients with complete data for the corresponding covariate set. The medication-adjusted model additionally included baseline beta-blocker, ACEI/ARB/ARNI, and statin use and was restricted to patients with complete cardiovascular medication records.

ACEI, angiotensin-converting enzyme inhibitor; ARB, angiotensin receptor blocker; ARNI, angiotensin receptor -neprilysin inhibitor; CI, confidence interval; eGFR, estimated glomerular filtration rate; HER2, human epidermal growth factor receptor 2; IHC, immunohistochemistry; LVEF, left ventricular ejection fraction; NSAI, non-steroidal aromatase inhibitor; OR, odds ratio; SD, standard deviation; SII, systemic immune-inflammation index; SIRI, systemic inflammation response index.

### 3.9 Subgroup analyses showed generally consistent associations across clinically relevant strata

As shown in Table 3, the associations of baseline SII and SIRI with study-defined composite cardiac dysfunction were generally consistent across subgroups defined by age, hypertension status, and renal function. For both biomarkers, the adjusted ORs remained above 1.0 across all strata, and no statistically significant interactions were observed for age, hypertension status, or eGFR (all *P* for interaction >0.05). The associations remained statistically significant in patients aged <65 and ≥65 years, in those with and without hypertension, and in those with eGFR <90 and ≥90 mL/min/1.73 m^2^.

**Table 3:** Subgroup analyses of the associations of baseline SII and SIRI with study-defined composite cardiac dysfunction.

Subgroup	Category	*N*	Events, *n*	Adjusted OR per 1-SD increase (95% CI)	*P* for interaction
Panel A, SII (per 1 SD increase in In-transformed SII)					
Age-years					0.181
	< 65	128	23	3.198 (1.630, 6.274)	
	≥ 65	141	40	1.847 (1.235, 2.763)	
Hypertension					0.795
	No	194	37	2.090 (1.334, 3.274)	
	Yes	75	26	2.727 (1.451, 5.125)	
eGFR, mL/min/1.73m^2^					0.652
	< 90	78	20	2.704 (1.256, 5.821)	
	≥ 90	191	43	2.069 (1.399, 3.058)	
Panel B. SIRI (per 1 SD increase in In-transformed SIRI)					
Age-years					0.243
	< 65	128	23	2.590 (1.361, 4.926)	
	≥ 65	141	40	1.658 (1.104, 2.489)	
Hypertension					0.46
	No	194	37	2.076 (1.312, 3.283)	
	Yes	75	26	1.889 (1.047, 3.408)	
eGFR, mL/min/1.73m^2^					0.564
	< 90	78	20	2.999 (1.380, 6.518)	
	≥ 90	191	43	1.801 (1.202, 2.699)	

ORs are reported per 1-SD increase in natural log-transformed SII or SIRI. Subgroup-specific estimates were derived from models adjusted for age, NSAI type, observation duration, hypertension, diabetes mellitus, history of cardiovascular disease, baseline LVEF, and eGFR, excluding the variable used to define the corresponding subgroup where appropriate. *P* values for interaction were obtained by including a multiplicative interaction term between the inflammatory index and the subgroup variable.

SII, systemic immune-inflammation index; SIRI, systemic inflammation response index; OR, odds ratio; CI, confidence interval; SD, standard deviation; NSAI, non-steroidal aromatase inhibitor; LVEF, left ventricular ejection fraction; eGFR, estimated glomerular filtration rate.

### 3.10 Exploratory internal-validation analyses suggested additional information from baseline SII and SIRI

To further evaluate whether baseline SII and SIRI could support pretreatment risk stratification in a predictive phenotyping framework, exploratory machine-learning analyses were performed using baseline-only feature sets. Post-treatment LVEF, post-treatment BNP, LVEF change, BNP change, and other follow-up-derived variables were excluded from all feature sets to avoid outcome leakage.

In stratified five-fold random forest analyses with fold-wise preprocessing, the base clinical model yielded an AUC of 0.704 (95% CI 0.633–0.767), average precision of 0.410, and Brier score of 0.188. After adding SII, the AUC was 0.782 (95% CI 0.721–0.840), average precision was 0.552, and Brier score was 0.159. After adding SIRI, the corresponding values were 0.764 (95% CI 0.691–0.827), 0.468, and 0.166. The model including both markers yielded an AUC of 0.773 (95% CI 0.708–0.834), average precision of 0.506, and Brier score of 0.162 (Figure 7).

**Figure 7:**
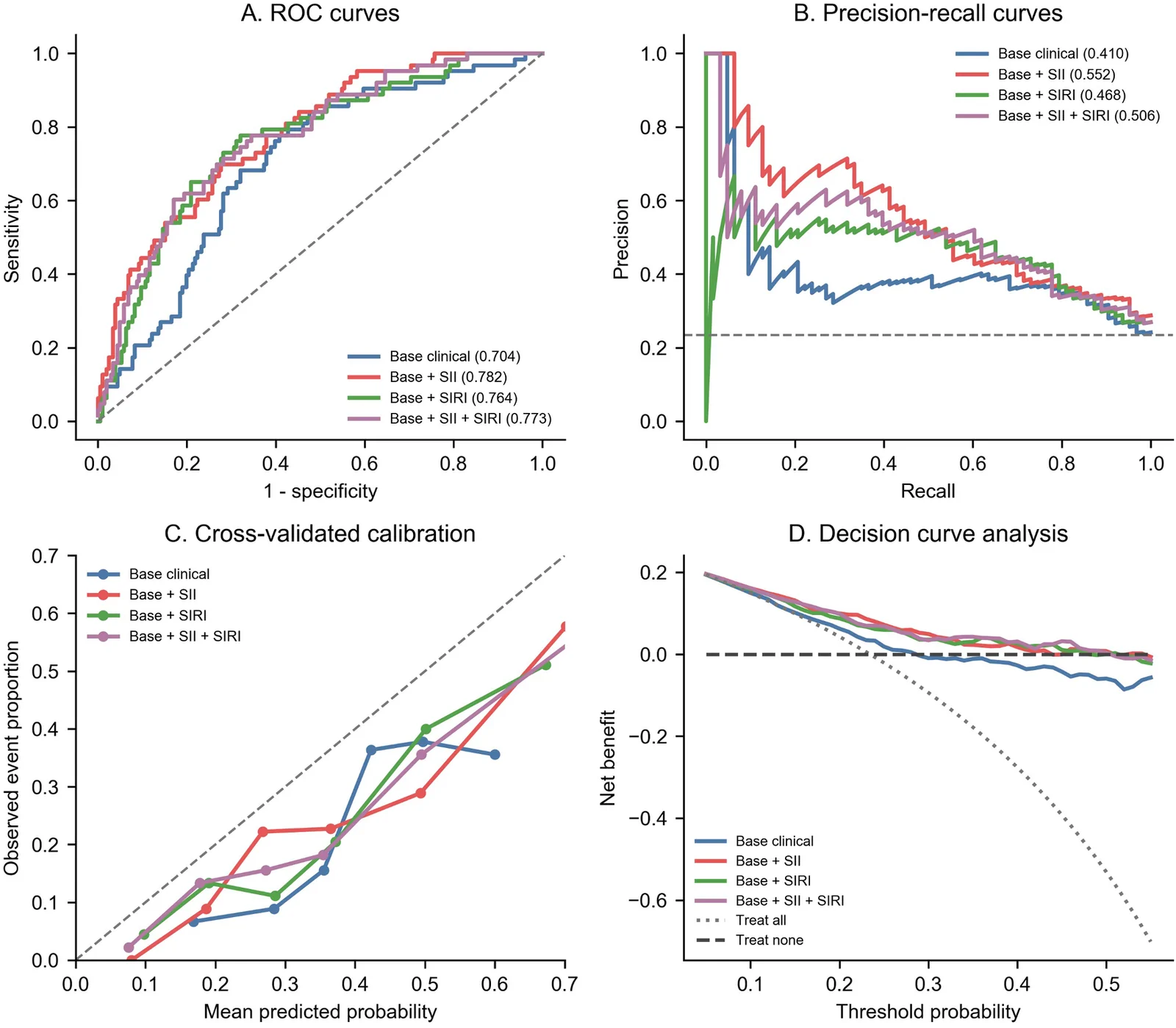
Exploratory machine-learning performance using stratified five-fold cross-validation with fold-wise preprocessing. **(A)** ROC curves. **(B)** Precision-recall curves. **(C)** Calibration curves. **(D)** Decision-curve analysis of random forest models using the base clinical feature set alone or with SII, SIRI, or both indices. SII, systemic immune-inflammation index; SIRI, systemic inflammation response index; ROC, receiver operating characteristic; AUC, area under the receiver operating characteristic curve.

Across exploratory algorithms, inflammatory-index feature sets generally showed numerical discrimination gains over their corresponding base models, but calibration varied. For the primary random forest, calibration intercepts ranged from −0.740 to −0.649 and slopes from 1.021 to 1.177. Given the modest cohort size, the limited number of endpoint events, and the absence of external validation, these findings should be interpreted as exploratory and may remain subject to optimism despite internal cross-validation. External validation is required before clinical application.

## 4 Discussion

In this retrospective cohort, baseline SII and SIRI were independently associated with study-defined composite cardiac dysfunction during NSAI therapy. These associations remained significant in medication-adjusted and available-case oncology-adjusted sensitivity analyses. Exploratory internally cross-validated analyses showed numerical improvements in discrimination after the incorporation of SII and SIRI. However, these estimates were derived from a modest single-center cohort with only 63 endpoint events and should not be interpreted as stable or externally validated predictive performance.

The *post hoc* component-attribution analyses further showed that associations of SII and SIRI were stronger and statistically significant for the LVEF-attributed component, whereas the corresponding BNP-attributed estimates were weaker and nonsignificant. This pattern suggests that the overall association was not predominantly driven by the relatively nonspecific BNP-related component. However, the BNP analysis was based on a substantially smaller paired-measurement population, and the nonsignificant estimates should not be interpreted as evidence of no association.

This interpretation is biologically plausible in the clinical context of long-term NSAI therapy. These agents are administered almost exclusively to postmenopausal women, a population in whom estrogen deficiency, cardiovascular aging, and chronic low-grade inflammatory activation converge to create a more vulnerable cardiovascular background. Estrogen loss has been associated with endothelial dysfunction, adverse metabolic and vascular remodeling, oxidative stress, and amplification of inflammatory signaling, while aging is further associated with immunologic remodeling and inflammaging (11, 15, 20, 21). Within this framework, elevated SII and SIRI may capture a systemic inflammatory milieu in which estrogen deprivation, vascular aging, oxidative stress, endothelial dysfunction, and immune remodeling converge to amplify cardiac vulnerability during long-term NSAI therapy.

SII and SIRI can be understood as readily available surrogate markers of baseline immune-inflammatory burden (17). Rather than representing isolated hematologic abnormalities, these composite indices integrate multiple circulating cellular components involved in inflammatory activation, immune regulation, and tissue remodeling. In our cohort, both markers were independently associated with subclinical cardiac dysfunction, supporting the view that pre-treatment inflammatory status is clinically relevant to subsequent cardiac vulnerability during NSAI therapy. Notably, SII showed slightly stronger discrimination and greater decision-curve-based net benefit than SIRI. One possible explanation is that SII incorporates the platelet component in addition to neutrophils and lymphocytes, thereby capturing not only inflammatory activation but also a thrombo-inflammatory dimension that may be particularly relevant in postmenopausal estrogen-deprived states (22, 23). By contrast, SIRI may more strongly reflect monocyte-related innate immune activation and inflammatory remodeling (24, 25).

The prediction findings support exploratory interpretation. Fold-wise cross-validated baseline-only models showed numerical and statistically tested improvements in discrimination after adding SII or SIRI, while Brier scores and calibration also improved relative to the base model. The modest cohort size, 63 events, substantial raw eGFR missingness, and absence of external validation support hypothesis-generating use. NRI and IDI were reported as exploratory reclassification summaries.

The present findings also extend the existing literature on the cardiovascular safety of aromatase inhibitors in an important way. Prior population-based studies have primarily focused on overt cardiovascular outcomes and have linked aromatase inhibitors exposure to higher risks of heart failure, cardiovascular mortality, arrhythmia, and ischemic heart disease, particularly with prolonged treatment (12, 13, 26). However, those studies were not designed to identify early functional deterioration or to determine which patients are more vulnerable before clinically overt events occur. By contrast, the present findings focused on study-defined composite cardiac dysfunction as an earlier phenotype and further showed that baseline immune-inflammatory burden helps distinguish a subgroup of patients at higher risk. In this sense, the present work adds a susceptibility-based and risk-phenotyping perspective to the existing safety literature: rather than suggesting that NSAI exposure leads to uniform cardiac injury, our results support the view that clinically silent functional decline may preferentially develop in patients with a more pro-inflammatory baseline profile. The definition of the primary endpoint warrants careful interpretation. In the present study, study-defined composite cardiac dysfunction was used as a retrospective operational outcome rather than as a formal guideline-defined CTRCD category. The BNP-based component required both a relative increase of ≥40% and a follow-up concentration of ≥100 pg/mL. The relative-change criterion was intended to capture a substantial within-patient increase, whereas the absolute threshold reduced classification resulting solely from proportional increases from very low baseline values. Although the individual thresholds were informed by previous BNP and breast-cancer cardiotoxicity literature, their combined use has not been externally validated as an age-adjusted or standardized cardio-oncology endpoint. Importantly, the associations of SII and SIRI remained directionally consistent when the absolute BNP threshold was increased to 125, 150, 200, and 400 pg/mL, suggesting that the findings were not solely dependent on the 100-pg/mL cutoff. Nevertheless, these analyses were *post hoc* and do not establish an optimal BNP threshold (27, 28). Similarly, the LVEF component was intended to capture a substantial longitudinal decline in systolic function but did not additionally require a specific follow-up LVEF range and therefore should not be interpreted as a formal CTRCD severity category (4). Serial GLS was not routinely available and could not be reliably reconstructed retrospectively, precluding application of contemporary GLS-based criteria for mild asymptomatic CTRCD. Accordingly, the composite outcome should be interpreted as an exploratory, study-defined measure of early cardiac functional or biomarker deterioration within the context of this retrospective cohort.

The clinical importance of the present findings lies in the possibility of integrating immune-inflammatory profiling into baseline cardiovascular risk assessment during NSAI therapy. Current surveillance strategies are largely guided by conventional demographic and clinical factors, but such approaches may not adequately capture biological susceptibility to subclinical cardiac dysfunction (29). In this context, SII and SIRI are attractive because they are inexpensive, routinely available, and measurable before treatment initiation. Their value may lie not in replacing standard cardiovascular evaluation, but in refining it by identifying patients with a heightened immune-inflammatory background who may merit closer monitoring during long-term endocrine therapy (30). From a practical cardio-oncology perspective, patients with high baseline inflammatory burden, particularly those in the upper range of SII or SIRI and those with concomitant age-related or cardiovascular risk factors, may represent a subgroup in whom closer echocardiographic and biomarker follow-up should be considered to facilitate earlier detection of clinically silent functional decline.

An available-case comparison was performed to assess potential selection bias. Baseline LVEF, the principal leukocyte components, NLR, MLR, SII, and SIRI were generally comparable between included and excluded patients. Baseline BNP was available in only a small and potentially selectively tested subset of excluded patients. Moreover, because most excluded patients were identified from outpatient screening records and did not enter the standardized study data-collection workflow, BMI, cardiovascular comorbidities, medication use, and NSAI type were not systematically available. These missing data were not interpreted as the absence of the corresponding conditions. Therefore, the comparison should be regarded as an available-case assessment, and residual selection bias cannot be excluded.

Several limitations should be acknowledged. First, this was a single-center retrospective study with a modest sample size, and residual confounding cannot be excluded. Although an included-versus-excluded comparison was performed, it was restricted to a limited set of available variables and could not evaluate cardiovascular risk factors, NSAI type, medication use, or several oncologic and clinical characteristics. Substantial residual selection bias therefore remains possible. No patient in the included cohort had received anthracycline-based chemotherapy; therefore, cumulative anthracycline dose was not applicable. Nevertheless, detailed non-anthracycline chemotherapy regimens and cumulative doses, radiotherapy dose and laterality, and overall oncologic treatment burden were not uniformly captured. Residual confounding from these prior or concurrent cancer treatments therefore remains possible. Accordingly, the available-case oncology-adjusted model should be interpreted as a partial sensitivity analysis rather than as complete control of treatment-related confounding. In addition, the small number of anastrozole-treated patients and endpoint events precluded reliable drug-specific subgroup analyses.

Second, the primary endpoint was a study-defined composite of LVEF- and BNP-based deterioration rather than a formal guideline-defined CTRCD category or a clinical cardiovascular outcome. The BNP criterion has not been specifically validated in older postmenopausal women receiving NSAI therapy. BNP may be influenced by age, renal function, atrial fibrillation, other comorbidities, and assay-related variation. Although age and eGFR were included in Model 3 and associations remained directionally consistent at higher BNP thresholds, residual biomarker-related confounding and endpoint misclassification cannot be excluded. Moreover, the LVEF criterion did not require a specific follow-up LVEF range. The operational and heterogeneous nature of this composite endpoint should therefore be considered when interpreting the findings, and longer follow-up is needed to determine whether it is associated with subsequent heart failure, cardiovascular hospitalization, or cardiovascular mortality.

Third, echocardiographic measurements were obtained from routine clinical reports rather than from centralized core-laboratory reassessment. Although LVEF was measured using two-dimensional transthoracic echocardiography and the biplane Simpson method, information on reader identity, blinding, and inter- or intra-observer reproducibility was unavailable. Given the inherent variability of LVEF measurement, a decline of 10 percentage points may partly reflect measurement variability, particularly in patients with preserved baseline LVEF or values near the endpoint threshold. GLS was not routinely acquired or reported, and the archived examinations were insufficient for reliable retrospective strain analysis; therefore, contemporary GLS-based criteria for mild asymptomatic CTRCD could not be applied.

Fourth, endpoint ascertainment relied on the last available routine-care LVEF or BNP assessment rather than on prospectively scheduled measurements. Variation in follow-up timing and testing frequency may therefore have influenced endpoint classification, although observation duration was included in the adjusted models.

Finally, the prediction analyses were based on internal cross-validation within a relatively small cohort with 63 endpoint events. Although all preprocessing, model fitting, and any data-driven feature selection were restricted to the training folds, five-fold cross-validation and the *post hoc* nested analysis remained entirely internal. The modest sample size and limited number of endpoint events resulted in wide uncertainty and a substantial risk of optimism. Therefore, the machine-learning findings should be regarded as exploratory supportive evidence rather than as validated prediction models, and external validation in independent cohorts is required.

In conclusion, higher baseline SII and SIRI were associated with study-defined composite cardiac dysfunction during NSAI therapy in postmenopausal women with hormone receptor-positive breast cancer. These associations and the internally estimated discrimination gains should be considered exploratory. The retrospective outcome definition and absence of external validation preclude interpretation as a validated CTRCD phenotype or support for clinical implementation.

## Data Availability

The original contributions presented in the study are included in the article/Supplementary Material, further inquiries can be directed to the corresponding authors.
