# Supplementary material for: Baseline SII and SIRI identify immune-inflammatory susceptibility and improve risk stratification of early subclinical cardiac dysfunction during non-steroidal aromatase inhibitor therapy
Source: Front Cardiovasc Med. 2026 Oct 9;13:1888328. doi: 10.3389/fcvm.2026.1888328 (PMC13691644; doi:10.3389/fcvm.2026.1888328)
Supplement: Supplementary file 1 [file Datasheet1.docx]

**Supplementary Figure S1. Post hoc sensitivity analyses using progressively higher follow-up BNP thresholds.**

(A) Alternative-composite event counts at BNP thresholds of 100, 125, 150, 200, and 400 pg/mL. Blue bars represent primary events retained independently of the BNP threshold, and orange bars represent additional BNP-only events retained at each cutoff. The 100-pg/mL reference analysis retained all 63 primary events. (B) Fully adjusted Model 3 ORs and 95% CIs per 1-SD increase in z-lnSII and z-lnSIRI at each threshold. These analyses assessed sensitivity to progressively stricter absolute BNP thresholds and were not intended to determine an optimal diagnostic cutoff.

| **Variable** | **Available, n** | **Missing, n (%)** | **Data-handling procedure** |
| --- | --- | --- | --- |
| Hypertension | 247 | 22 (8.2%) | MICE using logistic regression |
| Diabetes mellitus | 245 | 24 (8.9%) | MICE using logistic regression |
| History of cardiovascular disease | 242 | 27 (10.0%) | MICE using logistic regression |
| eGFR | 99 | 170 (63.2%) | Recalculated from available source measurements |
| Platelet count | 259 | 10 (3.7%) | MICE using predictive mean matching |
| Neutrophil count | 260 | 9 (3.3%) | MICE using predictive mean matching |
| Lymphocyte count | 260 | 9 (3.3%) | MICE using predictive mean matching |
| Monocyte count | 260 | 9 (3.3%) | MICE using predictive mean matching |

**Supplementary Table S1. Variable-specific missingness and data-handling procedures**

Missingness was assessed before statistical imputation. Values obtained through source-record verification or deterministic recalculation were not considered statistically imputed. Remaining missing covariate data used in the primary association analyses were handled using multiple imputation by chained equations. Cardiovascular medication and selected oncologic variables were analyzed using available-case datasets.

MICE, multiple imputation by chained equations; SII, systemic immune-inflammation index; SIRI, systemic inflammation response index; LVEF, left ventricular ejection fraction; eGFR, estimated glomerular filtration rate; BNP, B-type natriuretic peptide.

| **Variable** | **Included patients (n=269)** | **Excluded patients (n=89)** | **P value** |
| --- | --- | --- | --- |
| Age, years | 64.6 ± 9.0 | 66.8 ± 9.4 | 0.057 |
| Baseline LVEF, % | 66.4 ± 4.5 | 64.7 ± 5.8 | 0.117 |
| Neutrophil count, ×10^9^/L | 3.07 (2.14, 4.13) | 2.98 (2.33, 3.88) | 0.924 |
| Lymphocyte count, ×10^9^/L | 1.67 (1.27, 2.12) | 1.72 (1.33, 2.19) | 0.501 |
| Monocyte count, ×10^9^/L | 0.38 (0.30, 0.50) | 0.38 (0.30, 0.49) | 0.838 |
| NLR | 1.66 (1.27, 2.68) | 1.66 (1.28, 2.48) | 0.972 |
| MLR | 0.22 (0.17, 0.30) | 0.22 (0.17, 0.29) | 0.764 |
| SII | 396.2 (270.7, 645.9) | 331.0 (253.0, 488.1) | 0.142 |
| SIRI | 0.7 (0.4, 1.2) | 0.67 (0.39, 1.04) | 0.986 |

**Supplementary Table S2. Available-case comparison of baseline characteristics between included and excluded patients**

Data are presented as mean ± SD or median (IQR), as appropriate. Comparisons were based on observed data without statistical imputation, and variable-specific denominators are reported. Normally distributed variables were compared using the Welch t test, and non-normally distributed variables were compared using the Mann-Whitney U test.

NLR, neutrophil-to-lymphocyte ratio; PLR, platelet-to-lymphocyte ratio; MLR, monocyte-to-lymphocyte ratio; SII, systemic immune-inflammation index; SIRI, systemic inflammation response index; LVEF, left ventricular ejection fraction; BNP, B-type natriuretic peptide; NSAI, non-steroidal aromatase inhibitor.

| **Variable** | **Included patients (n=199)** | **Excluded patients (n=70)** | **P value** |
| --- | --- | --- | --- |
| Study-defined composite cardiac dysfunction | 50/199 (25.1%) | 13/70 (18.6%) | 0.326 |
| Age, years | 63.63 ± 9.03 | 67.20 ± 8.22 | 0.003 |
| Hypertension | 55/199 (27.6%) | 20/70 (28.6%) | 0.878 |
| Diabetes mellitus | 23/199 (11.6%) | 11/70 (15.7%) | 0.404 |
| Hyperlipidemia | 11/199 (5.5%) | 8/70 (11.4%) | 0.108 |
| History of cardiovascular disease | 13/199 (6.5%) | 3/70 (4.3%) | 0.769 |
| BMI, kg/m^2 | 24.01 ± 3.46 | 23.91 ± 3.45 | 0.825 |
| Systolic blood pressure, mmHg | 129.54 ± 14.05 | 132.86 ± 17.93 | 0.163 |
| Diastolic blood pressure, mmHg | 77.92 ± 9.12 | 74.79 ± 10.58 | 0.029 |
| eGFR, mL/min/1.73 m^2 | 91.61 ± 15.21 | 91.30 ± 12.07 | 0.862 |
| SII | 522.27 ± 418.44 | 535.54 ± 411.06 | 0.817 |
| SIRI | 1.02 ± 1.30 | 1.40 ± 2.16 | 0.176 |

**Supplementary Table S3. Characteristics of patients included in and excluded from the partial oncology-adjusted sensitivity analysis**

The partial oncology-adjusted sensitivity analysis required complete data for all nine selected oncology covariates. Patients with missing data for any covariate were excluded. Continuous variables are presented as mean ± SD or median (IQR), and categorical variables as n/N (%). SMD, standardized mean difference.
